# Movement Behaviors in Youth on the Autism Spectrum: The HUNT Study, Norway

**DOI:** 10.1007/s10803-025-06835-7

**Published:** 2025-04-21

**Authors:** Ingrid Okkenhaug, Terese Wilhelmsen, Paul Jarle Mork, Ingar Mehus

**Affiliations:** 1 Department of Sociology and Political Science, Norwegian University of Science and Technology, Trondheim, Norway; 2 Department of Educational Science, University of South-Eastern Norway, Drammen, Norway; 3 Department of Public Health and Nursing, Norwegian University of Science and Technology, Trondheim, Norway

**Keywords:** HUNT, 24-hour movement behavior, Physical activity, Sedentary behavior, Screen time, Autism

## Abstract

Research consistently show that autistic youth are less physically active compared to their neurotypical peers. However, there is limited understanding of how gender influences physical activity (PA) patterns among neurodiverse youth compared to the general population. This study aims to examine 24-hour movement behaviors - PA, sedentary behavior (SB), and sleep duration - among autistic youth (*n* = 71) in Norway, in comparison to peers with Attention-Deficit/Hyperactivity Disorder (ADHD) (*n* = 411) and the general youth population (*n* = 3805). The data is from the Young-HUNT4 study, linked with diagnostic information from the Norwegian Patient Registry. Variables explored are objective accelerometer-measured PA, SB, and sleep duration, self-reported participation in organized and unorganized physical activities, and screen activities. Results confirms that autistic youth engage in lower levels of moderate-to-vigorous PA, while demonstrating similar levels of light PA. They also spend more time sitting and comparable time sleeping. Autistic youth participate less in sport and were less likely to use commercial gyms. However, their participation in outdoor activities were similar to their peers. Regarding screen activities, autistic youth spent more time playing video games, while youth with ADHD were more engaged in social media. Among autistic youth, the only gender difference found was in video games. In conclusion, autistic youth are less physically active overall and spend significant time in SB. However, their comparable participation in light PA suggests opportunities for promoting further participation. Additionally, exergaming could offer a promising avenue to increase PA in this population.

Research consistently show that autistic youth are less physically active (Jones et al., 2017; Liang et al., 2020; Lobenius-Palmér et al., 2018), spend less time sleeping (Liang et al., 2023), and more time engaging in screen activities compared to typical developed peers (Haegele et al., 2023; Healy et al., 2017; Must et al., 2013; Stiller & Mößle, 2018). Physical activity (PA), sedentary behavior (SB), and sleep duration have traditionally been studied separately. However, research is moving toward an integrated approach that considers the amount of time spent in each of these movement behaviors over a 24-hour period (Pedišić et al., 2017; Rosenberger et al., 2019). Accompanying 24-hour activity frameworks we find recommendations concerning each movement behavior, and the Canadian 24-hour movement guidelines (Tremblay et al., 2016) have been adopted by several countries. Recommendations for PA suggest that youth should accumulate at least 60 min/day of moderate-to-vigorous PA (MVPA), coinciding with the WHO guidelines for MVPA (WHO, 2020). Tremblay et al. (2016) recommends 8–10 h of uninterrupted sleep per night and restricting recreational screen activities to no more than 2 h/day. Guidelines for light PA (LPA) and sitting are less specific due to a lack of evidence, but it is recommended that youth get “several hours of a variety of structured and unstructured LPAs”, and “limit sitting for extended periods” (Tremblay et al., 2000, p. 324). Autistic youth are less likely to meet guidelines for PA and screen activities compared to youth in the general population (Healy et al., 2019, 2021; Li et al., 2022). Furthermore, studies on sleep duration have yielded varying findings, with some showing no difference and others indicating that autistic youth are less likely to meet recommended sleep guidelines (Healy et al., 2019, 2021).

Physical activity is a complexed phenomenon, and PA participation can include a variety of activities on different arenas and contexts. Little is known about the changes in and preferences of PA among autistic youth (Corder et al., 2013; van Sluijs et al., 2021). However, studies indicate that autistic youth are less likely to participate in organized sport (Healy et al., 2017; Mangerud et al., 2014; McCoy & Morgan, 2020; Stanish et al., 2017) and school-based PA and physical education seems to be particularly challenging arenas (Arnell et al., 2018; Gurkan & Kocak, 2021; Lamb et al., 2016; Obrusnikova & Miccinello, 2012). To our knowledge, research on such activity patterns is scarce in the Scandinavian context.

Although research interest on autistic youth’s participation in PA has increased the last two decades (Okkenhaug et al., 2024), there are still important knowledge gaps. First, it is important to develop a better understanding of PA and SB among autistic youth compared to neurotypical and neurodiverse peers. Knowledge about what PA arenas autistic youth prefer or avoid is key to support a life-long physically active lifestyle. Second, few studies have explored possible gender differences in autistic youth’s PA participation. PA patterns among adolescents in the general population show consistent differences between boys and girls, in which boys are often found to be more physical active and with girls showing greater decline in PA with increasing age (Farooq et al., 2020; van Sluijs et al., 2021). However, the variation in PA explained by gender among autistic children and youth have shown inconsistent results and needs more research (Jones et al.,^2017^). This seems of special importance considering the number of children and youth diagnosed with autism spectrum disorder (ASD) has increased both in Norway and other high-income countries over the last 30 years (Chiarotti & Venerosi, 2020; Surén et al., 2012, 2019). The global prevalence is estimated to 1–2% (Lord et al., 2022; Zeidan et al., 2022). In Norway, the periodic prevalence of ASD among 2–17-year-old children is estimated to 0.8%, increasing to 1.3% in adolescents aged 15–17 years (Kiselev et al., 2020). On a global scale the male-to-female prevalence ratio is highly variable with males outnumbering females (Zeidan et al., 2022) and in Norway, the prevalence has been found to be three times higher among males than females from the age of four (Kiselev et al., 2020).

On this background, the overall aim of the present study was to explore gendered patterns of 24-hour movement behaviors (PA, SB and sleep duration) in autistic youth compared with two comparison groups: the general population and youth with Attention-Deficit/Hyperactivity Disorder (ADHD). To achieve this, we report (a) PA, SB and sleep duration measured by accelerometry, (b) self-reported participation in organized sport, unorganized PA, physical education, commercial gyms, and outdoors activity, and (c) self-reported time spent on screen activities, including screen-based entertainment, video games, and social media.

## Method

### Procedures and Participants

The current study is based on data from Young-HUNT4 linked with data on ASD and ADHD diagnoses from the Norwegian Patient Registry (NPR). The fourth survey of the Trøndelag Health Study (HUNT4) was carried out from 2017 to 2019, in which all adolescent (age 13–19 years) in the Nord-Trøndelag area in Norway (*n* = 10609) were invited to participate in Young-HUNT4 (http://www.ntnu.edu/hunt/young-hunt). Invitation and data collection was done during school hours, and participants completed a comprehensive questionnaire, a clinical examination and were asked to wear two accelerometers for one week. Of those invited, 8066 (76.0%) responded to the questionnaire, and of whom, 5664 (54.6%) wore accelerometers. Regarding the study area, Nord-Trøndelag is fairly representative of Norway in terms of the absence of large cities, economic structure, industry, sources of income, age distribution, and immigrant populations. (Holmen et al., 2014; Rangul et al., 2024; Åsvold et al., 2023). See Rangul et al. (2024) for cohort profile.

All HUNT data are linked to the unique personal identification number assigned to all Norwegian citizens. This allowed us to link individual data from the Young-HUNT4 study with ASD and ADHD diagnoses from the NPR. The NPR is an administrative database covering all specialist health-care services in Norway (Bakken et al., 2019). In the NPR, diagnoses are registered in accordance with the International Statistical Classification of Diseases and Related Health Problems version 10 (ICD 10) (WHO, 2016). All individuals registered with an ASD diagnosis (F84), except F84.2, F84.3 and F84.4 in accordance with Surén et al. (2013), in NPR were categorized accordingly and included in the autistic population in the current study (*N* = 71, 42.3% female, mean age 15.7 year, SD = 1.8). For comparison, individuals registered with the diagnosis of ADHD were included (*N* = 411, 47.2% female, mean age 15.9 years, SD 1.8). Participants in Young-HUNT4 who completed all of the included measures in the current study, and who were not diagnosed with ASD or ADHD, were included as a comparison group representative of the general population (*N* = 3805, 58.4% female, mean age 16.0 years, SD 1.8).

### Device Measured Physical Activity and Sedentary Behavior

Physical activity types (standing, walking, running, and cycling), SB (sitting and laying down), and sleep duration were measured for 7 days using two tri-axial AX3 accelerometers (Axivity, Ltd., Newcastle, United Kingdom). One accelerometer was placed centrally on the front of the right thigh and one centrally at the lower back at the third lumbar segment (L3). The details regarding attachment, configuration of the accelerometers, and processing of data have been described in detail elsewhere (Kongsvold et al., 2023). In brief, the AX3 OmGui software (version 1.0.0.37; Open Movement, Newcastle University, United Kingdom) was used to configure the sensors and the sensors were attached by trained nurses during school hours. After measurements were completed, the data were downloaded and segmented into 5 s windows and 161 features were computed for each window. These features were then fed into an eXtreme Gradient Boosting (XGBoost) machine learning model trained to predict lying, sitting, standing, slow walking (< 4 km/h), moderate walking (4.1 to 5.4 km/h), brisk walking (> 5.4 km/h), running, and cycling (Bach et al., 2022; Logacjov et al., 2021; Logacjov, Pedersen Ludvigsen, Logacjov et al., 2024a, b). Separate machine learning models were trained to predict no-wear time (Wold & Skaugvoll, 2019) and sleep duration (Logacjov, Skarpsno et al., 2024).

The sum of running, cycling, moderate walking, and brisk walking was considered MVPA. Slow walking and standing was considered LPA, and sitting down and lying down was considered SB (Ross et al., 2020). Only complete days with 24 h of measurements were included in the analysis, i.e., the days attaching and removing the accelerometers were omitted from the analysis. Additionally, only individuals with ≥ 3 days of measurement were included. We also excluded days with less than 5 min of walking to avoid including from days where participants were not wearing the accelerometers, and individuals with less than 60 min of average time per day sleeping.

### Self-Reported Physical Activity and Screen Activities

Participation in physical education was measured with a single question: How many school hours during a week do you participate in the physical education at school? The response options were: None, 1 h, 2 h, 3 h, 4 h, and 5 h or more (Rangul et al., 2024). Non-organized and self-organized forms of PA were measured by two questions: “How often do you usually partake in non-organized training with others?” and “How often do you usually exercise alone (on own initiative)?”. Response options were: Never, 2–3 times a month or less, once a week, 2–3 times a week, and 4 times a week (Rangul et al., 2024).

Other PA participation was measured by asking: “How often do you usually do the following exercise?”, with the following eight response options (choosing multiple options was allowed): (1) Team sports (e.g. football, volleyball, handball, ice hockey, squash), (2) Endurance sports (e.g. running, cross-country skiing, cycling, swimming), (3) Aesthetic sports (e.g. dance, gymnastics, aerobics), (4) Martial arts/combat sports (e.g. judo, karate, taekwondo, boxing, weightlifting), (5) Technical sports (e.g. riding, track sports, ski jumping, skateboarding), (6) Ski sports (e.g. snowboard, alpine), (7) Exercising at a fitness-center or gym, and (8) Outdoor life (e.g. hiking, cross-country skiing) (Rangul et al., 2024). For each activity the participants were asked to indicate the frequency using the same response options as for non-organized and self-organized PA, described above. Participation in individual sports is presented by a single variable, chosen by the single activity with highest self-reported attendance, among the following individual activities; endurance sports, aesthetic sports, martial arts/combat sports, technical sports, and ski sports.

Self-reported time spent on screen activities was measured with three duration questions from the WHO Health Behavior in School-Aged children (WHO-HBSC) (Currie et al., 2014) considering screen activities, differentiating between weekdays and weekends: “In your spare time, how many hours per day do you spend watching TV or other screen-based entertainment”, “In your spare time, how many hours per day do you play video games (on PC, game console, tablet, cell phone, etc.)” and “In your spare time, how many hours per day do you spend on social medias or surfing/chatting online”. The response options were: Not at all, less than half an hour a day, ½-1 h a day, 2–3 h a day, 4–6 h a day, and approximately 7 h or more a day. For this study, weekdays and weekends were merged. To investigate adherence to screen recommendations we based our analysis on the single screen activity with the highest self-reported engagement– the preferred screen activity.

### Statistical Analysis

Device measured, continuous, variables were tested for assumptions using parametric statistics, with several violations concerning normal distribution (Shapiro-Wilk test) and homogeneity of variance (Levine´s test). Especially the violation of homogeneity is problematic and calls for robust statistical tests. To deal with violation of homogeneity and unequal sample sizes, differences between the three groups (autism, ADHD and general population) were tested with Welch´s ANOVA. When indicating significant (*p* <.05) differences between groups, Fisher-Hayter post hoc tests were conducted for a closer investigation of what groups differed from each other and effect sizes were calculated and reported with partial eta squared (η_p_^2^). Partial eta squared statistics is useful when comparing the size of effects within a study (Fritz et al., 2012) and are often interpreted in terms of large (0.14), medium (0.06), and small (0.01) effects (Cohen, 1988). The unequal variance Welch´s t-test was used to test for gender differences. When indicating significant (*p* <.05) gender differences, Cohen´s *d* effects sizes were calculated and reported.

For self-reported, ordinal, variables of participation in PA arenas and screen activities, group differences were investigated using the Kruskal-Wallis test. When indicating significant (*p* <.05) differences between groups, post-hoc comparisons using Dunn’s method with a Bonferroni correction for multiple tests were conducted, and effect sizes were reported with eta squared (η^2^). Gender differences were investigated using the Mann-Whitney U-test (also known as Wilcoxon Rank Sum Test), and effect sizes (r) were calculated for significant (*p* <.05) gender differences.

### Ethics

In the Young-HUNT4 survey, all participants gave their informed written consent, for youth younger than 16 years, their guardian also provided written consent. The current study was approved by the Regional Committee for Medical and Health Research Ethics (REK, ref. 536870).

In line with the research aim, the results are presented in three sections: (1) device-measured PA, SB and sleep duration, (2) self-reported participation in PA arenas, and (3) self-reported screen activities.

### Device-Measured Physical Activity, Sedentary Behavior, and Sleep Duration

**Fig. 1:**
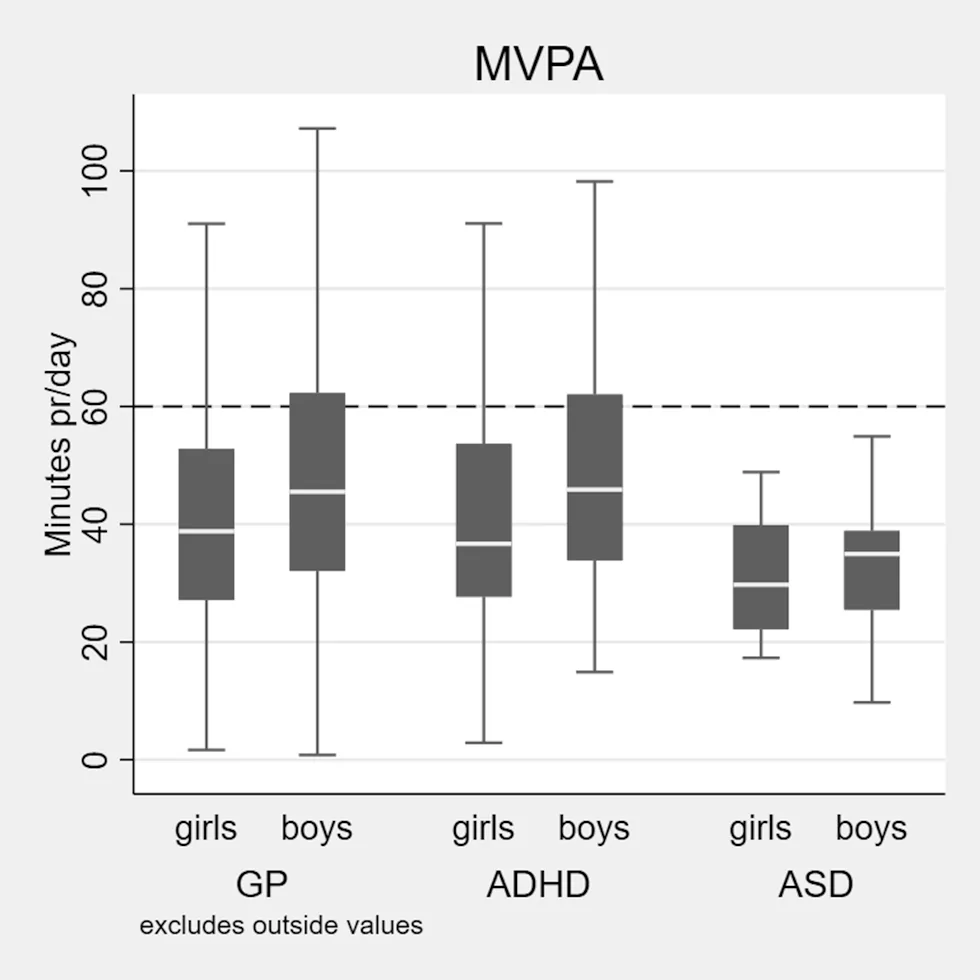
Boxplot of Moderate to Vigorous Physical Activity (MVPA) with dotted line indicating recommendation of minimum 60 min per day

As indicated in Table 1, autistic youth had lower device-measured MVPA compared to youth from the general population and youth with ADHD, with no difference between the two comparison groups (F(2, 95.58) = 22.83, *p* <.01, η_p_^2^ = 0.01). As shown in Fig. 1, none of the autistic youth achieved the recommended 60 min of MVPA per day. In addition to results in Fig. 1 we calculated the exact percentages for the three groups, showing that 0% of the autistic youth, 21.8% of the general population, and 23.4% of youth with ADHD achieved the recommendations. No between group differences were found concerning LPA of slow walking and standing.

**Table 1:** Mean (and SD) of device-measured physical activity, sedentary behavior and sleep duration (min/day) in the general youth population and youth diagnosed with ADHD and ASD

Behavior		GP^d^				ADHD^e^				ASD^f^			
	Total	Girls	Boys		Total	Girls	Boys		Total	Girls	Boys		
	(*N* = 3805)	(*N* = 2222)	(*N* = 1583)	t-values	(*N* = 222)	(*N* = 125)	(*N* = 97)	t-values	(*N* = 40)	(*N* = 20)	(*N* = 20)	t-values	f-values
MVPA^a^	44.76 (21.35)	41.89 (19.79)	48.80 (22.78)	-9.73**	44.59 (21.26)	40.36 (18.95)	50.05 (22.88)	-3.37**	32.68 (11.06)	31.66 (10.40)	33.70 (11.87)	− 0.58	22.83** ADHD = GP > ASD
LPA^b^: *Slow walking*	54.63 (19.35)	54.10 (17.57)	55.37 (21.57)	-1.94	52.77 (20.77)	50.09 (19.40)	56.22 (22.03)	-2.17*	49.83 (16.71)	49.60 (15.61)	50.07 (18.14)	− 0.09	2.40
*Standing*	214.76 (66.24)	229.10 (59.08)	194.64 (70.41)	15.89**	209.62 (82.53)	212.54 (63.87)	205.86 (101.92)	0.56	214.93 (65.80)	229.81 (72.82)	200.05 (55.85)	1.45	0.41
SB^c^: *Sitting*	443.73 (106.35)	435.86 (93.64)	454.77 (121.15)	-5.20**	412.52 (117.61)	419.83 (109.66)	403.09 (127.09)	1.03	498.51 (124.72)	479.12 (121.95)	517.89 (127.54)	0.98	11.41** ASD > GP > ADHD
*Lying down*	253.29 (132.93)	242.62 (120.54)	268.26 (147.34)	-5.70**	298.39 (161.09)	286.99 (154.03)	313.07 (169.44)	-1.18	208.84 (118.09)	205.04 (129.38)	212.63 (108.87)	− 0.20	11.30** ADHD > ASD = GP
Sleep duration	428.82 (45.15)	436.42 (40.31)	418.16 (49.26)	12.14**	422.11 (48.91)	430.19 (39.28)	411.70 (57.61)	2.71**	435.21 (44.91)	444.77 (49.80)	425.66 (38.30)	1.36	2.42

Notes

^a^ Moderate to Vigorous Physical Activity

^b^ Light Physical Activity

^c^ Sedentary Behavior

^d^ General Population

^e^ Attention-Deficit/Hyperactivity Disorder

^f^ Autism spectrum disorder

*p <.05, **p <.01

Concerning SB, we found differences between all three groups on sitting (F(2, 90.71) = 11.41, *p* <.01, η_p_^2^ = 0.003), with autistic youth sitting more compared to both comparison groups, and youth with ADHD sitting less compared to the general population. Additionally, youth with ADHD were found to be lying down while awake more than the general population and autistic youth (F(2, 91.36) = 11.30, *p* <.01, η_p_^2^ = 0.003).

**Fig. 2:**
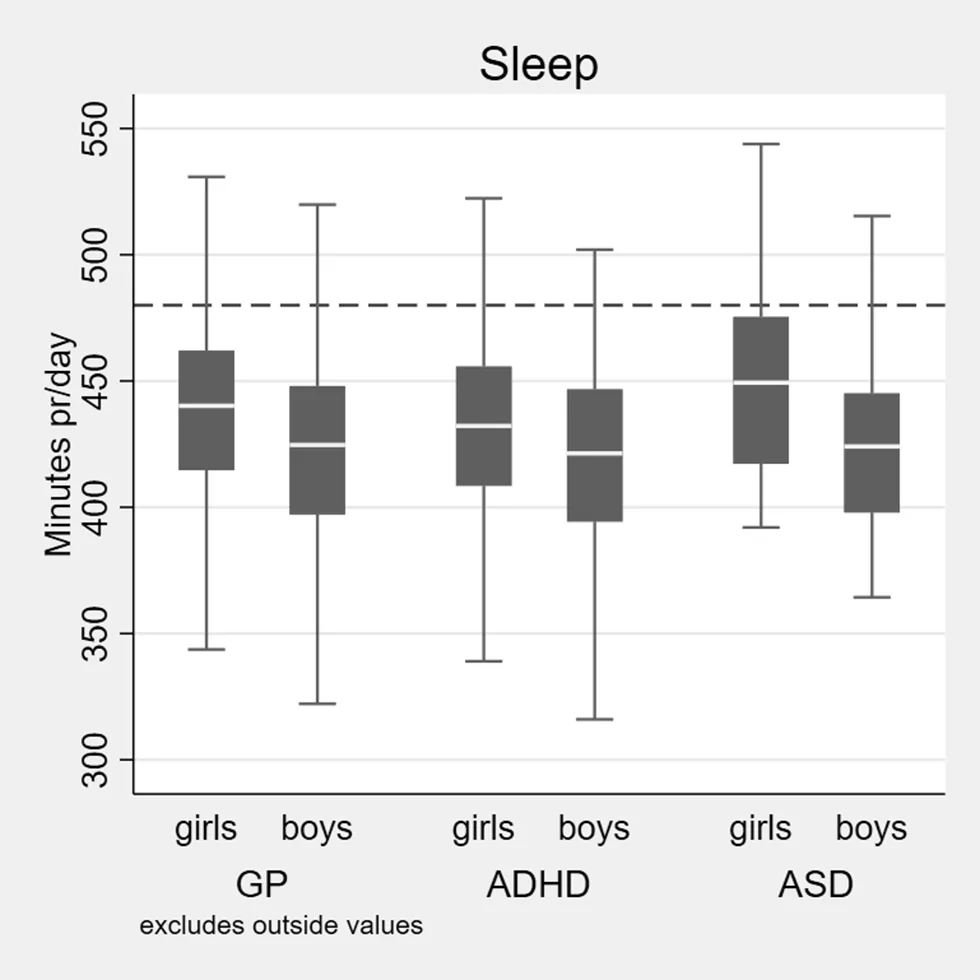
Boxplot of Sleep duration with dotted line indicating recommendation of minimum 480 min per day

For sleep duration there were no differences between the three groups. Looking at the recommendations for sleep in Fig. 2, relatively few in all three groups achieved the recommended amount of 480 min per day. Calculating the exact percentages showed that 8.8% of the general population, 8.1% of youth with ADHD, and 12.5% of autistic youth achieved the recommendations.

Also note that while there were no differences between boys and girls among autistic youth on any of the device-measured parameters, there were observed differences between boys and girls in both comparison groups. In the general population, boys (M = 48.80, SD = 22.78) had higher MVPA compared to girls (M = 41.89, SD = 19.79), (t(3103.37)=-9.73, *p* <.01, *d* = 0.33). Boys also sat down (M = 454.77, SD = 121.15) and lay down (M = 268.26, SD = 147.34) more than girls (M = 435.86, SD = 93.64), (t(2849.27)=-5.20, *p* <.01, *d* = 0.18); (M = 242.62, SD = 120.54), (t(2971.05)=-5.70, *p* <.01, *d* = 0.19). Whereas girls stood more (M = 229.10, SD = 59.08) and slept more (M = 436.42, SD = 40.31) compared to boys (M = 194.64, SD = 70.41), (t(3026.64) = 15.89, *p* <.01, *d* = 0.54); (M = 418.16, SD = 49.26), (t(2971.48) = 12.14, *p* <.01, *d* = 0.41). For youth with ADHD, boys had higher MVPA (M = 50.05, SD = 22.88) and slow walking (M = 56.22, SD = 22.03) compared to girls (M = 40.36, SD = 18.95), (t(186.53)=-3.37, *p* <.01, *d* = 0.47); (M = 50.09, SD = 19.40), (t(194.21)=-2.17, *p* <.05, *d* = 0.30). Whereas girls (M = 430.19, SD = 39.28) slept more compared to boys (M = 411.70, SD = 57.61), (t(162.78) = 2.71, *p* <.01, *d* = 0.38).

### Self-Reported Participation on Different Physical Activity Arenas

The general population reported higher participation in physical education compared to both neurodiverse groups (c^2^(2) = 19.70, *p* <.01, η^2^ = 0.005), as shown in Table 2. The general population reported being more active in team sports compared to both neurodiverse groups, and youth with ADHD being more active compared to autistic youth (c^2^(2) = 72.30, *p* <.01, η^2^ = 0.017). This is the highest effect size when comparing participation in the three groups across the different PA arenas. This corresponds with the high non-participation in team sports among autistic youth, as indicated by a median of 1 (never participating). For individual sports, the general population reported higher participation compared to both neurodiverse groups (c^2^(2) = 19.17, *p* <.01, η^2^ = 0.005).

**Table 2:** Median (and 25th;75th percentile) of self-reported participation on different physical activity arenas in the general youth population and youth diagnosed with ADHD and ASD

Physical activity arenas		GP^b^				ADHD^c^				ASD^d^			
	Total	Girls	Boys	z	Total	Girls	Boys	z	Total	Girls	Boys	z	χ^2^
Physical education	3 (3;4)	3 (3;4)	3 (3;5)	-6.21**	3 (3;4)	3 (3;3.5)	3 (3;4)	-2.56**	3 (3;3)	3 (3;3)	3 (3;4)	− .38	19.70** GP > ADHD = ASD
Team sports	3 (1;4)	3 (1;4)	4 (1;4)	-3.40**	1 (1;4)	1 (1;4)	1 (1;4)	-1.87	1 (1;3)	1 (1;3)	1 (1;3)	.70	72.30** GP > ADHD > ASD
Individual sports	3 (2;4)	3 (2;4)	3 (2;4)	2.00*	3 (1;4)	3 (1;4)	2.5 (1;4)	.10	2 (1;4)	2 (1;4)	2 (1;4)	.82	19.17** GP > ASD = ADHD
Nonorganized PA^a^ with others	3 (1;4)	2 (1;3)	3 (2;4)	-6.25**	2 (1;3)	2 (1;3)	2 (1;4)	-1.79	1 (1;2)	1 (1;3)	1 (1;2)	.70	50.48** GP > ADHD > ASD
Personal training	3 (2;4)	3 (2;4)	3 (2;4)	− .53	3 (1;4)	2 (1;4)	3 (1;4)	− .89	2 (1;4)	2 (1;3)	1 (1;4)	1.20	46.01** GP > ADHD = ASD
Gym	1 (1;3)	1 (1;3)	1 (1;3)	-1.98*	1 (1;3)	1 (1;3)	1 (1;4)	-2.29*	1 (1;1)	1 (1;1)	1 (1;1)	-1.15	23.74** GP = ADHD > ASD
Outdoors	2 (1;3)	2 (1;3)	2 (1;2)	6.07**	2 (1;2)	2 (1;2)	2 (1;2)	.01	2 (1;3)	2 (1;3)	2 (1;3)	− .11	9.33* GP > ADHD

Notes

^a^ Physical activity

^b^ General Population

^c^ Attention-Deficit/Hyperactivity Disorder

^d^ ASD Autism spectrum disorder

*p <.05, **p <.01

Autistic youth were less active in non-organized PA with others compared to both comparison groups, (c^2^(2) = 50.48, *p* <.01, η^2^ = 0.012). Here we find similar trends for non-participation as for team sport, with a median of 1 among autistic youth. For personal training alone the general population reported higher participation than both neurodiverse groups (c^2^(2) = 19.17, *p* <.01, η^2^ = 0.011).

Commercial gyms were the least preferred PA arena among autistic youth with both comparison groups reporting more participation (c^2^(2) = 23.74, *p* <.01, η^2^ = 0.006). Here, non-participation is relatively high in all three groups, while for autistic youth, the 25th and 75th percentiles show consistent non-participation. Outdoor PA were the only arena where autistic youth was equally active as both comparison groups (c^2^(2) = 9.33, *p* <.01, η^2^ = 0.002).

Again, while there were no gender differences in patterns of participation on PA arenas among autistic youth, there were clear differences between boys and girls in the general population, with boys reporting higher participation in physical education (z=-6.21, *p* <.01, *r* =.10), team sports (z=-3.40, *p* <.01, *r* =.06), non-organized PA with others (z=-6.25, *p* <.01, *r* =.10), and going to the gym (z=-1.98, *p* <.01, *r* =.03). Girls on the other hand report higher participation in individual sports (z = 2.0, *p* <.05, *r* =.03) and outdoors activities (z = 6.07, *p* <.01, *r* =.10). Among youth with ADHD, boys reported higher participation in physical education (z=-2.55, *p* <.01, *r* =.13) and going to the gym (z=-2.29, *p* <.01, *r* =.12).

### Self-Reported Screen Activities

**Fig. 3:**
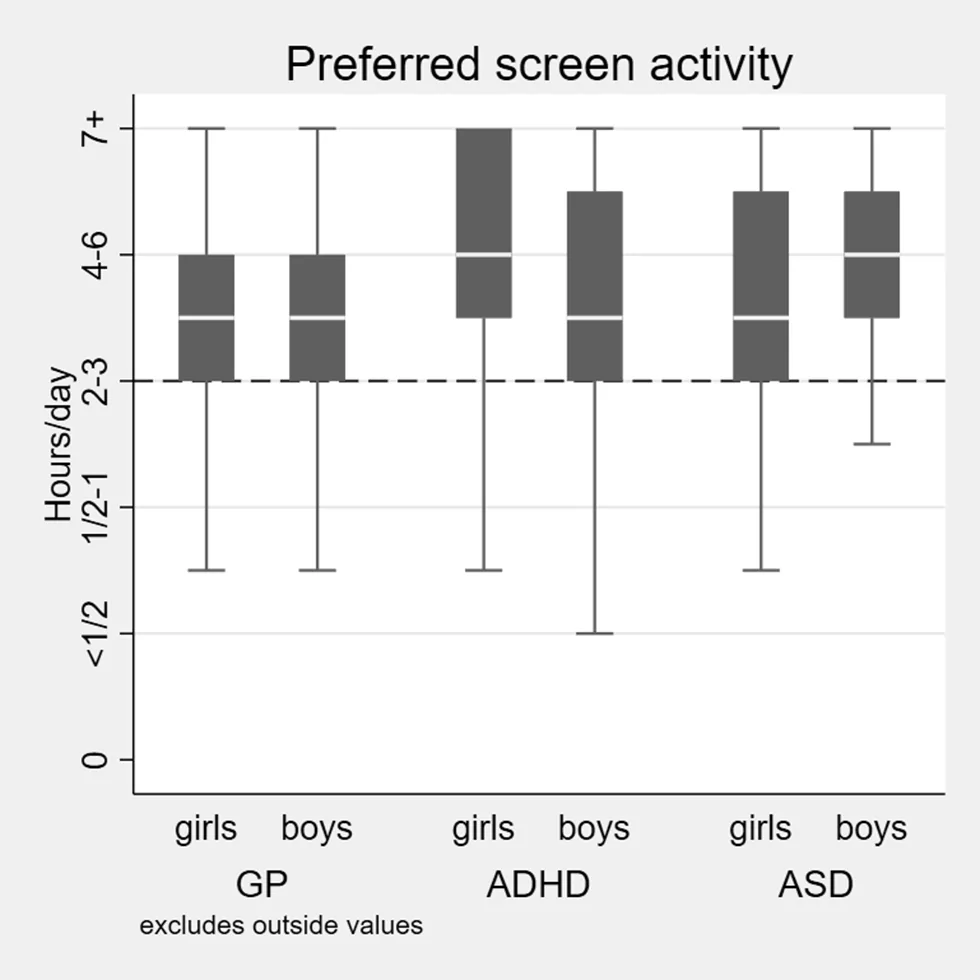
Boxplot of preferred screen activity (out of screen-based entertainment, video-games and social media), with dotted line indicating recommended dosage of maximum 2 h per day

As indicated in Table 3 the general population reported less time used on screen-based entertainment than both neurodiverse groups (c^2^(2) = 7.06, *p* <.05, η^2^ = 0.002). While there were no gender differences among autistic youth, girls reported more time on screen-based entertainment than boys in the general population (z=-2.22, *p* <.05, *r* =.04) and the ADHD group (z=-2.29, *p* <.01, *r* =.12). Autistic youth reported spending more time playing video games than both comparison groups (c^2^(2) = 51.78, *p* <.01, η^2^ = 0.01), and boys reported spending more time on video games compared to girls in all three groups; general population (z=-21.85, *p* <.01, *r* =.35), ADHD (z=-4.03, *p* <.01, *r* =.21), and autistic (z=-2.06, *p* <.05, *r* =.26). Autistic youth report to spend the least time on social media, while youth with ADHD spend the most time (c^2^(2) = 51.78, *p* <.01, η^2^ = 0.004). Girls reported spending more time on social media compared to boys in the general population (z = 15.37, *p* <.01, *r* =.25) and in the ADHD group (z = 4.63, *p* <.01, *r* =.24) with no gender differences among autistic youth. Regarding the screen recommendation of a maximum of two hours per day, Fig. 3 shows that, regardless of population, most youth exceed this limit in a single screen activity every day. Calculating the exact percentages showed that 17.7% of the general population, 14.4% of youth with ADHD, and 13.4% of the autistic youth spent less than two hours per day on their preferred screen activity.

**Table 3:** Median (and 25th;75th percentile) of self-reported screen activities in the general youth population and youth diagnosed with ADHD and ASD

Screen activities		GP^a^				ADHD^b^				ASD^c^			
	Total	Girls	Boys	z	Total	Girls	Boys	z	Total	Girls	Boys	z	c^2^
Screen-based entertainment	3.5 (3;4)	3.5 (3;4)	3.5 (3;4)	2.22*	3.5 (3;4.5)	4 (3;4.5)	3.5 (2.5;4.5)	2.29*	4 (3;5)	4 (3; 5)	4 (2.5;5)	0.16	7.06* ASD = ADHD > GP
Video-games	3.5 (2;4.5)	2.5 (1.5;4)	4 (3;4.5)	-21.85**	4 (2;5)	3.5 (2;5)	4.5 (3;5)	-4.03**	4.5 (3;5)	4 (3; 4.5)	4.5 (4;5.5)	-2.06*	51.78** ASD = ADHD > GP
Social media	4 (3.5;5)	4.5 (3.5;5)	4 (3;4.5)	15.37**	4.5 (3;5.5)	4.5 (4;5.5)	4 (3;5)	4.63**	3.5 (2;4.5)	4 (2.5;5.5)	3.5 (2;4)	1.47	17.20** ADHD > GP > ASD

Notes

^a^ General Population

^b^ Attention-Deficit/Hyperactivity Disorder

^c^ Autism spectrum disorder

*p <.05, **p <.01

## Discussion

To the best of our knowledge this is the first study in the Norwegian context to map and compare movement behaviors of neurodivergent youth with the general population in a 24-hour activity cycle. Firstly, we would like to highlight some similarities that seems to characterize youth in general. (1) Youth are not getting enough sleep, in our sample, the average youth get 7 h of sleep, which is well below the recommended 8–10 h (Tremblay et al., 2016). (2) Youth in general are not physically active enough, with only 21.7% of the total sample achieving the recommended minimum of 60 min of MVPA per day. And (3), youth are sitting and lying down for extended periods of time, with the two SB together accounting for an average of 11.5 h a day. These general trends are supported by previous research (Hao et al., 2024; Rollo et al., 2020). Looking at arenas for PA, autistic youth engage in roughly the same activities as both comparison groups, but the frequency of participation varies, consistent with previous research (Stanish et al., 2017).

Replicating previous research (Jones et al., 2017; Liang et al., 2020; Menear & Ernest, 2020; Rech et al., 2022), autistic youth in our sample show lower levels of MVPA than both comparison groups, they also have lower adherence to the PA guidelines. Previous research on adherence to PA guidelines present a mixed picture, with our findings supporting low adherence (Li et al., 2022; Lobenius-Palmér et al., 2018; Menear & Ernest, 2020; Stanish et al., 2017). An important finding is the similar levels of LPA across the three study groups, the aspect of LPA has to a lesser extent than MVPA been explored in previous research. Lobenius-Palmér et al. (2018) found lower levels of LPA in autistic youth compared to the general population, while Haegele et al. (2021) found no significant difference in average daily LPA.

For SB the between group patterns are more complex, autistic youth sit more than both comparison groups but lay down less than youth with ADHD and more than the general population. To explain these differences, we suggest combining device-measured SB with the types of screen activities the groups self-report to engage in. Suggesting that different screen activities encourage different postures, such as video games being easier to engage in while sitting, while social media can be as easy to explore laying down. Higher engagement in screen activities among autistic youth is supported by previous research (Healy et al., 2017; Mazurek et al., 2012; Menear & Ernest, 2020; Must et al., 2013). Lower engagement with social interactive media (i.e. social media) than solitary screen-based media (television/IPAD and video games) is further supported by Mazurek et al. (2012) and Stiller and Mößle (2018), and can be related to social or communicative expectations, although we do not know the nature of the video games played by participants in the current study. Autistic youths’ engagement in video games has in some cases successfully been used to enhance PA, through different forms of exergaming (Graham et al., 2022; Obrusnikova & Cavalier, 2011), this could be a potential way to enhance PA in this population.

Given the known evidence of the positive health outcomes of PA (Biddle et al., 2019; Bull et al., 2020), and especially MVPA, it is concerning that autistic youth achieve a substantial lower level of PA than recommended. In line with this, it is crucial to further investigate which arenas for PA autistic youth prefers, also including the structure and organization of these arenas to further facilitate PA.

### Participation in Physical Activity Arenas

Autistic youth in the current study participate less in physical education, regardless of the subject being mandatory throughout primary and secondary school in Norway. Considering that participating in physical education seems to be positively related to PA participation outside school hours (Bremer et al., 2020; Obrusnikova & Miccinello, 2012), an important goal is to ensure participation in physical education. The importance of participation in school-based PA was reaffirmed in a systematic literature review by Liang et al. (2020), which indicated that school settings accounted for the most time engaged in MVPA among autistic youth.

Previous research have found autistic youth to report low participation in organized sport in general (Healy et al., 2017; Mangerud et al., 2014; McCoy & Morgan, 2020; Stanish et al., 2017), these findings are replicated in the current study. Additionally, we were able to distinguish between team- and individual sports, finding that autistic youth report higher participation in individual sports compared to team sports. Autistic youths differing levels of participation between sports could indicate different opportunities for adaptation and experiencing an inclusive environment in some sports compared to others. Participation in non-organized PA follows a similar pattern, with autistic youth participating less in non-organized activity with others and individually. A possible explanation could be that autistic youth experience challenges with initializing PA as shown in previous research (Arnell et al., 2018; Hilton et al., 2008). Further, the higher rates of non-participation among autistic youth can partially be explained with the barriers they might experience when engaging in PA (Arkesteyn et al., 2023; Okkenhaug et al., 2024).

In Norway, the PA arena of commercial gyms have increased in popularity over the past decades contributing to a trend where youth discontinue their participation in organized sport to continue being physically active at the gym (Seippel & Skille, 2018). Interestingly, the results show this arena to be the least favored among autistic youth. In previous research on autistic youths experiences from the gym, gym facilities have been described as unorganized, characterized by self-organized PA, and loud which could contribute to overwhelming sensory experiences (Blagrave et al., 2021; Healy et al., 2013; Obrusnikova & Miccinello, 2012). The low compatibility between commercial gyms and autism characteristics can be one explanation for low participation in this specific PA arena among autistic youth.

Outdoor PA was identified as the only PA arena where autistic youth are equally active as their peers. Outdoor PA can be characterized as low-threshold activities, such as hiking, cross-country skiing, camping etc., often done in family- or friend units, which could contribute to the similar participation rates across populations. Similarly, Stanish et al. (2017) found that walking/hiking was the most reported activity among autistic youth. Furthermore, previous research has indicated that normative performance standards of organized sport and physical education could function as a barrier for autistic youths’ participation (Okkenhaug et al., 2024). Outdoor PA appear to be a relevant alternative for activity without set movement patterns, measures for performance, or competition.

### Gender Differences

The results indicated clear gender differences in both PA levels and patterns in the general population and youth with ADHD. These findings support consistent gendered patterns found in the general population where boys are more active compared to girls. For autistic youth we found no gender differences in neither PA level nor participation. These findings echo previous research exploring differences between autistic boys and girls (Healy et al., 2017; Jones et al., 2017; McCoy & Morgan, 2020). For example, the study by Healy et al. (2017) found no difference in MVPA, LPA or sport participation between autistic boys and girls. In the present study, the gendered patterns of screen activities were also more profound in both comparison groups than for autistic youth. For autistic youth the only gender difference was that boys played more video games compared to girls.

One plausible explanation for limited gender differences in PA patterns among autistic youth is that autistic boys and girls experience similar restrictions in their PA participation explained by core autistic features independent of gender, such as sensory sensitivities, preferences for certainty and challenges with social communication (Okkenhaug et al., 2024). Another plausible explanation could be autistic youth’s perspectives on and experiences with gendered PA expectations in society and personal identity. Nonetheless, gendered PA patterns among autistic youth have received limited research attention and further research is needed to understand the non/gendered patterns observed among autistic youth better. For example, qualitative research exploring autistic youth’s experience with how PA participation intersect with personal preferences and gendered identities could provide much needed insights.

### Strengths and Limitations

This study has several strengths, including a population-based sample of youth covering 76% of the total adolescent population in Nord-Trøndelag, still, the risk of self-selection bias is present, especially in the autistic and ADHD population. Linkage to the NPR ensured that we only included individuals officially diagnosed with ASD or ADHD. There is a lack of consensus in the accelerometer literature regarding practices such as measurement periods, data treatment, and analysis (De Craemer & Verbestel, 2021; Hedayatrad et al., 2020), making comparisons across studies difficult. Nevertheless, the trends in PA levels are similar, regardless of accelerometer type. The accelerometer data does not allow for differentiation between, e.g. school hours and leisure. This could have added nuance to our understanding of PA and SB.

As measures of PA arenas and screen activities were self-reported, the included variables are susceptible to information bias. Screen habits develop and change quickly; hence the current relevance of the screen variables can be questioned. Nevertheless, the use of comparison groups to identify similarities and differences in both PA and screen activities is highly relevant.

### Implications

Mapping movement behavior in a 24-hour activity framework show some interesting similarities and differences when comparing neurodiverse youth with the general population. The groups are similar in terms of sleep and LPA, so the differences are found in MVPA and SB. Autistic youth, are less active and spend more time sitting, and our data suggest that they spend a substantial amount of their sitting time engaging in screen activities. The obvious implication would be to reduce time spent on screen activities and increase MVPA. However, a general advice of increasing MVPA without considering the suitability of different PA arenas could end up counterproductive. Results show that autistic youth report particularly low participation in team sports, non-organized PA with others and commercial gyms. This could implicate that autistic youth find these PA arenas less suitable for their needs and therefore choose to participate in individual sports, personal training and outdoor PA to a greater extent. Knowledge of the characteristics of such arenas might help parents, teachers and coaches to guide autistic youth in the right direction. On the other hand, one could argue that this is insufficient, suggesting that PA arenas should actively strive to better accommodate and include autistic youth by adapting to their needs and preferences.

Another implication is to utilize the potential of LPA, which has clear health benefits compared to SB (Poitras et al., 2016; Rosenberger et al., 2019). Additionally, our results suggest that combining LPA with outdoor PA is especially welcomed by autistic youth. LPA could also be effectively combined with exergaming. Autistic boys spend the most time playing video games, and research has already shown this to be a promising way of increasing PA among autistic youth (Graham et al., 2022; Obrusnikova & Cavalier, 2011).

## Data Availability

The Trøndelag Health Study (HUNT) has invited persons aged 13–100 years to four surveys between 1984 and 2019. Comprehensive data from more than 140,000 persons having participated at least once and biological material from 78,000 persons are collected. The data are stored in HUNT databank and biological material in HUNT biobank. HUNT Research Centre has permission from the Norwegian Data Inspectorate to store and handle these data. The key identification in the data base is the personal identification number given to all Norwegians at birth or immigration, whilst deidentified data are sent to researchers upon approval of a research protocol by the Regional Ethical Committee and HUNT Research Centre. To protect participants’ privacy, HUNT Research Centre aims to limit storage of data outside HUNT databank and cannot deposit data in open repositories. HUNT databank has precise information on all data exported to different projects and are able to reproduce these on request. There are no restrictions regarding data export given approval of applications to HUNT Research Centre. For more information see: http://www.ntnu.edu/hunt/data.
